# Protocol for conducting a scoping review on the influence of low-stakes assessment on student learning in medical education

**DOI:** 10.12688/f1000research.158552.1

**Published:** 2024-12-19

**Authors:** Imran Zafar, Lamburt Schuwirth, Susan Waller, Carvalho Filho, Mohi Eldin Magzoub

**Affiliations:** 1 Medical Education, College of Medicine and Health Sciences, United Arab Emirates University, Al Ain, Abu Dhabi, United Arab Emirates; 2 Faculty of Medicine, Flinders University, Adelaide, South Australia, Australia; 3 Medical Education, University of Groningen, Groningen, The Netherlands

**Keywords:** Scoping Review, Low Stakes Assessments

## Abstract

**Rationale and Background:**

Low-stakes assessments enhance student learning outcomes by providing a comprehensive view of performance and promoting competency-based education. Multiple low-stakes assessments encourage continuous learning and incorporate formative feedback for a realistic and long-term environment. Diverse assessment methods ensure psychometric rigor, utility and a programmatic assessment approach with multiple data points affording high-stakes decisions on progression.

**Objective:**

This scoping review aims to systematically identify and map existing literature on the use of low-stakes assessments in medical education and determine their impact on student learning outcomes in terms of, motivation and engagement achievements/competencies.

**Methods and Analysis:**

The review will use the Joanna Briggs Institute’s framework for scoping review studies, searching eight databases and grey literature. A presearch will be done in PubMed, Scopus, and Google Scholar using terms related to low-stakes assessment, formative assessment, continuous assessment, and programmatic assessment within the context of medical education. The Covidence Systematic Review tool will aid in screening and conflict resolution.

The reference lists of included studies will be checked manually for other relevant literature. Two research team members will independently screen and extract data, resolving discrepancies with a third team member. Inclusion and exclusion criteria will be refined iteratively based on key research themes.

The review will follow PRISMA-P guidelines, focusing on the impact of low-stakes assessment on student learning in medical education.

**Ethics and dissemination:**

No ethical approval is required as all data will be collected from published and grey literature. Findings will be disseminated at relevant conferences and submitted for publication in peer-reviewed journals.

## Introduction

### Definition of low-stakes assessment in medical education:

Low-stakes assessments (LSAs) in medical education are evaluation methods with minimal impact on a student’s final grade or certification outcomes. These assessments are primarily used for formative purposes, providing opportunities for students to practice, receive feedback, and learn from mistakes without significant consequences ( Shrivastava & Shrivastava, 2022b). They are designed to monitor progress, identify learning gaps, and help both learners and educators focus on areas needing improvement ( Pastor et al., 2019; Schüttpelz-Brauns et al., 2018). Despite their low stakes, these assessments can influence student behavior and effort, particularly when performance is discussed with mentors or when there are consequences for non-participation ( Govaerts, 2014). To enhance effectiveness, it’s important that students perceive these assessments as valuable and relevant to their future practice, which can increase engagement and effort ( Schüttpelz-Brauns et al., 2018) ( Price et al., 2024). However, variability in students’ test-taking effort can affect validity, necessitating strategies to identify and exclude low-effort responses for more accurate results ( Wang et al., 2020; Schut et al., 2018). Low-stakes assessments contribute to building a longitudinal representation of student performance, are useful for continuous feedback, improving student feedback literacy, self-evaluation and learning, LSAs help reduce the stress and anxiety associated with high-stakes evaluations, creating a supportive learning environment ( Shrivastava & Shrivastava, 2022b). Overall, they are essential for continuous learning and improvement in medical education without the high pressure of significant academic consequences.

### What are the types of low-stakes assessment in medical education?

LSAs provide ongoing feedback and help identify students’ strengths and challenges, thereby guiding future learning and teaching strategies ( Abu-Zaid, 2013). Examples of LSAs are simulated clinical scenarios, objective structured clinical examinations (OSCEs), and questionnaires or written assessments, which are often used to evaluate non-technical skills ( Gordon et al., 2019). Additionally, low-stakes progress tests, such as those measured by the Test-taking Effort Short Scale (TESS), help gauge students’ effort and intrinsic motivation during assessments ( Schüttpelz-Brauns et al., 2018). Team-based learning (TBL) assessments, including Individual Readiness Assurance Tests (IRAT) and Group Readiness Assurance Tests (GRAT), also fall under low-stakes assessments, providing a structure for continuous feedback and performance tracking ( Vegi et al., 2022). Furthermore, workplace-based assessments like Direct Observation of Procedural Skills (DOPS), Mini-Clinical Evaluation Exercises (mini-CEX), and Case-based Discussions (CbD) are integral to assessing clinical competence in real-world settings, promoting active learning and immediate feedback ( Liu, 2012, Ref, 2024). The use of multiple-choice questions (MCQs) from the first year of medical education is another form of low-stakes assessment that helps students prepare for professional exams and promotes deep learning ( Wang et al., 2020). Peer assessments, such as the mini-peer assessment tool (mini-PAT), allow students to evaluate each other, fostering a collaborative learning environment and enhancing performance through peer feedback ( Shrivastava & Shrivastava, 2022a). Finally, Entrustable Professional Activities assess by the level of entrustment can be considered as types of low-stakes assessment. ( Kinnear et al., 2021). These diverse methods collectively contribute to a comprehensive and supportive assessment framework in medical education, balancing the need for evaluation with the goal of fostering a deep, sustained learning experience.

### How do low-stakes assessments engage medical students?

LSAs offer multiple benefits for medical students, enhancing their learning environment and academic experience. These individual assessments, which do not carry significant consequences, encourage students to engage more deeply with the material without the pressure of high-stakes exams. They guide the learning process and monitor educational programs, despite potential high variation in test-taking effort ( Price et al., 2024). Strategies like reviewing low test performance with mentors exploring the student’s perception of performance and offering constructive feedback and implementing consequences for non-participation can increase serious test-taking behavior, thereby improving the validity of these assessments ( Schüttpelz-Brauns et al., 2020).

LSAs foster self-regulated learning (SRL) and co-regulated learning (CRL), crucial for developing clinical reasoning, doctor-patient communication, and self-reflection skills ( Pastor et al., 2019). Detailed asynchronous feedback on low-stakes quizzes significantly improves exam performance, allowing students to review and synthesize content deeply outside the classroom ( Shrivastava & Shrivastava, 2022a). These assessments help identify patterns in student performance, predictive of future academic success, and inform targeted remediation interventions ( Schüttpelz-Brauns et al., 2018). Tools like the Test-taking Effort Short Scale (TESS) measure test-taking effort in LSAs, ensuring results reflect students’ true abilities by identifying and allowing exclusion of students results who are not making effort ( Price et al., 2024). Peer assessments, such as the mini-peer assessment tool (PAT), enhance learning outcomes by providing diverse feedback and fostering a collaborative learning environment ( Wang et al., 2020).

LSAs are less stressful, reducing the anxiety and pressure that can negatively impact learning outcomes. They increase student participation and engagement, as students are more likely to take part in assessments that do not significantly affect their grades or future. These assessments provide more opportunities for feedback and improvement, crucial in medical education for developing necessary skills and competencies. ( Shrivastava & Shrivastava, 2022b; Ontong, 2021).

The formative nature of LSAs provides regular feedback and opportunities to adjust learning strategies, helping students develop a growth mindset and focus on continuous improvement ( Ontong, 2021). Using various assessment methods, including LSAs, offers a comprehensive evaluation of students, reduces cheating likelihood, and includes engaging and effective assessments like quizzes, discussions, and case studies. Since they do not significantly impact final grades, low-stakes assessments reduce pressure, allowing students to focus on learning and improvement rather than just achieving high grades. They also provide flexibility and reversibility, enabling teachers to adapt their assessment strategies as needed ( Shrivastava & Shrivastava, 2022b; Ontong, 2021).

Overall, LSAs play a complementary role in supporting continuous learning and skill development in medical education ( Bains et al., 2023; Ganesan et al., 2023). They promote a culture of continuous learning, reduce stress and anxiety, and provide more opportunities for feedback and improvement.

### Scope and theoretical framework

A theoretical framework is essential for understanding the influence of LSAs on student learning in medical education.

Self-Determination Theory (SDT) is a suitable theoretical framework for investigating the influence of LSAs on student learning in medical education. SDT emphasizes autonomy, competence, and relatedness as key factors in motivating individuals, aligning with the importance of self-regulated learning (SRL) in medical training ( Neufeld, 2023, Ganesan et al., 2023).

To apply Self-Determination Theory (SDT) to low-stakes assessments in medical education, it is important to consider how these assessments can support students’ basic psychological needs of autonomy, competence, and relatedness; ( Neufeld, 2023). Providing students with choices in the format and timing of LSAs can promote a sense of autonomy ( Dutt et al., 2023). Involving students in the development of assessment criteria and rubrics fosters a sense of ownership ( Ganotice et al., 2023), and encouraging students to set their own learning goals and use assessments to monitor their progress enhances their autonomy ( Neufeld, 2023). Designing LSAs that are challenging yet achievable supports students’ feelings of competence ( Dutt et al., 2023). Providing timely and constructive feedback that focuses on progress and mastery rather than comparison to others, along with offering opportunities for students to demonstrate their knowledge and skills in various contexts, further reinforces competence ( Ganotice et al., 2023; Dutt et al., 2023; Neufeld, 2023). Creating a supportive and collaborative learning environment where students feel safe to take risks and learn from mistakes during low-stakes assessments addresses the need for relatedness ( Ganotice et al., 2023). Encouraging peer feedback and group discussions around assessment results fosters a sense of community and belonging ( Neufeld, 2023), while ensuring that assessments are aligned with the overall learning objectives and perceived as relevant and meaningful by students ( Dutt et al., 2023). Incorporating these SDT principles into the design and implementation of LSAs, medical educators can create a learning environment that supports students’ intrinsic motivation, engagement, and well-being ( Ganotice et al., 2023; Hirsh et al., 2024).

## Methods

### Research Questions

Self-Determination Theory (SDT) can guide our research questions related to how various factors in LSAs influence students’ basic psychological needs (autonomy, competence, and relatedness) and their subsequent motivation, engagement, and learning outcomes in educational settings ( Dutt et al., 2023; Netcoh, 2017). Below are some potential research questions that can be explored using SDT as a theoretical framework:

1. What are the implementation strategies of low-stakes assessment, in relation to the methods used, application and challenges?
2. How do different forms of low-stake assessment impact students’ learning in terms of motivation, engagement, and autonomy?
3. How do low-stakes assessment impact student achievement and competence?
4. How do students perceive and experience low-stakes assessments with reference to their influence on autonomy, competence, relatedness, and well-being compared to high- stakes summative assessment methods? ( Netcoh, 2017).

### Inclusion/Exclusion Criteria

To conduct this scoping review on how LSAs influence student learning in medical education, the following are the planned inclusion and exclusion criteria:

### Inclusion Criteria

1. Study Type: The review will include studies that are published in the form of articles, theses, or conference proceedings, both peer reviewed and published, unpublished studies and grey literature.
2. Topic: The primary focus of the study should be on the impact of LSAs on student learning in medical education.
3. Study Population: The studies should involve medical students, interns or residents (junior doctors in training) as the primary participants.
4. Assessment Type: The assessment should be classified as low-stakes, meaning it does not summatively (terminally) affect the student’s grade or academic standing, having less significant progress consequences.
5. Publication Date: The studies should be published after January 2000.
6. Language: The studies should be published in English to facilitate efficient data extraction and analysis.
7. Methodology: The studies can employ various research methods, including quantitative, qualitative, or mixed methods approach. Grey literature may include …

### Exclusion Criteria

1. High-Stakes Assessments: Studies that focus on high-stakes assessments, which significantly impact student grades or academic standing, will be excluded.
2. Non-Medical Education: Studies conducted in non-medical education settings or involving non-medical students will be excluded.
3. Non-English Language: Studies published in languages other than English will be excluded to ensure efficient data extraction and analysis.
4. Non-Primary Focus on Student Learning: Studies that do not explicitly investigate the impact of low-stakes assessments on student learning will be excluded.
5. Non-Medical Education Settings: Studies conducted in non-medical education settings, such as nursing or allied health, will be excluded.
6. Studies published or grey literature dated before 2000.

### Context

The context of this scoping review would be the influence of low-stakes assessments on student learning in medical education, focusing on the broader aspects of assessment methods and their impact on student learning outcomes.

The review aims to investigate how LSAs, which do not significantly impact student grades or academic standing, affect the learning process and outcomes of medical students. It will examine various types of LSAs, including online assessments, e-assessments, computer-assisted assessments, and portfolio-based assessments etc.

The review will focus on undergraduate medical education, as well as postgraduate medical education, such as residency programs. It will provide a comprehensive overview of the current literature on the challenges and opportunities associated with LSAs in medical education, including the role of technology and the impact on student learning outcomes

### Study Types

A wide range of study types are recommended to conduct this scoping review. Quantitative research like randomized controlled trials, quasi-experimental studies, cohort studies, cross-sectional studies, and pre-post studies should be covered. In order to gain insights into experiences and perspectives incorporating qualitative methods such as interviews, focus groups, and case studies are also important. A more comprehensive understanding can be obtained by mixed-methods studies that combine both quantitative and qualitative data. In addition, systematic reviews and meta-analyses will help summarize the existing research and highlight overall trends and gaps, while surveys can capture broader trends and opinions. Including such a wide range of study types makes sure that the topic is understood in all of its complexity.

The scoping review proposal will follow the Joanna Briggs Institute (JBI) methodology for scoping reviews and will be documented following the guidelines of the Preferred Reporting Items for Systematic Reviews and Meta-analyses extension for Scoping Reviews. It is planned to commence the review in September 2024, with an anticipated completion date in December 2024.

### Database Selection

The following key databases will be searched: PubMed, MEDLINE, ERIC, PsycINFO, Scopus, and Web of Science.

### Search Strategy

A comprehensive and iterative search strategy will be developed with assistance from a medical librarian. The following databases will be systematically searched:

• **PubMed**: To cover biomedical literature related to medical education.
• **MEDLINE**: For peer-reviewed studies on medical assessments and learning outcomes.
• **ERIC (Education Resources Information Center)**: To capture studies focused on educational practices and assessments in higher education.
• **PsycINFO**: For literature on psychological aspects of learning, motivation, and assessment.
• **Scopus**: To retrieve multidisciplinary studies, ensuring broad coverage.
• **Web of Science**: For high-impact articles on medical education and low-stakes assessments.
• **Google Scholar**: To capture grey literature and additional unpublished studies.

Additional grey literature will be searched through databases like **ProQuest Dissertations & Theses Global** and **OpenGrey** to include relevant theses, dissertations, and other non-peer-reviewed materials.

Keywords and **Medical Subject Headings (MeSH)** terms relevant to the topic will be employed, such as “low-stakes assessment,” “formative assessment,” “medical education,” “student learning,” and “programmatic assessment.” Boolean operators (AND, OR, NOT) will be used to refine the search results. The reference lists of included studies will be manually checked for additional relevant literature.

Screening Process and Iterative Refining of Criteria

An iterative process will be followed during the screening of titles and abstracts to ensure all relevant studies are captured. Two reviewers will independently screen the search results at two levels: title/abstract screening followed by full-text screening. The inclusion and exclusion criteria will be applied initially to a subset of studies to pilot test their adequacy.

During this process, if new themes or patterns emerge that require adjustment, the inclusion and exclusion criteria may be refined iteratively. For instance, if a certain type of low-stakes assessment appears to be underrepresented or newly relevant literature emerges during screening, these criteria will be adjusted accordingly to capture the breadth of available literature.

Any discrepancies between the two reviewers will be discussed, and if necessary, a third reviewer will be consulted to resolve conflicts. The team will maintain a record of all decisions made during the screening process to ensure transparency and reproducibility of the review **.**

### Data Extraction and Charting

Data from included studies will be extracted using a standardized data extraction form. This form will capture key study characteristics, including the study design, participant details, type of low-stakes assessment used, and outcomes related to student learning (e.g., motivation, engagement, competence).

The extracted data will be charted and summarized using tables and diagrams to provide an overview of the existing literature. Data will be presented in:

• Tables: Summarizing study characteristics (e.g., author, year, sample size, type of LSA).
• Diagrams or Flowcharts: Mapping the frequency and distribution of different types of low-stakes assessments across various studies and highlighting common themes.

A thematic analysis will be conducted to identify recurring themes and patterns across the studies. This analysis will be supported by a narrative synthesis, providing a descriptive summary of the findings related to the impact of low-stakes assessments on student learning in medical education. The results will be aligned with the objectives of the scoping review, and key findings will be visualized in diagrams or flowcharts where appropriate.

### Analysis

The analysis of the data will involve a descriptive synthesis to summarize and present the findings from the included studies. The following steps will be undertaken:

Categorization of LSAs: Types of LSAs identified in the studies will be categorized and described. This will provide an overview of the various assessment methods used in medical education.

Thematic Analysis: A thematic analysis ( Braun & Clarke, 2006) will be conducted to identify common themes and patterns related to the benefits, drawbacks, and impacts of low-stakes assessments on student learning. This will involve coding the extracted data and grouping similar concepts into themes.

Comparative Analysis: A comparative analysis will be performed to examine differences and similarities in the findings across studies. This will help to identify trends and variations in the use and impact of LSAs.

Identification of Best Practices: The review will highlight effective strategies and best practices for implementing low-stakes assessments in medical education. These will be derived from the successful approaches reported in the included studies.

Gap Analysis: A gap analysis will be conducted to identify areas where further research is needed. This will involve highlighting gaps in the existing literature and suggesting potential directions for future studies.

The results of the analysis will be presented in a narrative format, supported by tables and figures where appropriate. This will provide a comprehensive overview of the current state of knowledge on the influence of low-stakes assessments on student learning in medical education, as well as insights into best practices and future research directions.

### Ethics and consent

Ethical approval and consent were not required.

## Data Availability

No data are associated with this article. Reporting guidelines Figshare: Protocol for conducting a scoping review on the influence of low-stakes assessment on student learning in medical education, https://doi.org/10.6084/m9.figshare.27619560 ( Zafar et al., 2024). This project contains the following underlying data:
• PRISMA-P-checklist.docx Data are available under the terms of the Creative Commons Zero “No rights reserved” data waiver (CC0 1.0 Public domain dedication).
