# Supplementary material for: Protocol for conducting a scoping review on the influence of low-stakes assessment on student learning in medical education
Source: F1000Res. 2024 Dec 19;13:1537. [Version 1] doi: 10.12688/f1000research.158552.1 (PMC11890273; doi:10.12688/f1000research.158552.1)
Supplement: Peer review history

# Reviewer report: Reviewer response for version 1

**DOI:** 10.5256/f1000research.174161.r364640

**Published:** 2025-03-08

**Authors:** Tracey Taylor

**Affiliations:** 1 Oakland University William Beaumont School of Medicine, Rochester, USA

The manuscript submission entitled “Protocol for conducting a scoping review on the influence of low-stakes assessment on student learning in medical education” is a protocol for a scoping review examining the influence of low-stakes assessments (LSAs) on student learning in medical education. This manuscript submission describes the protocol to be used to perform a scoping review to investigate low-stakes assessment and its influence on medical student learning. LSAs are evaluations with minimal impact on final grades that provide formative feedback to students without significant consequences in terms of their grades/marks. The protocol will follow the Joanna Briggs Institute's framework and PRISMA-P guidelines, searching eight databases for literature published after 2000. The research questions focus on LSA implementation strategies, impact on student motivation and autonomy, effects on achievement, and student perceptions compared to high-stakes assessments. Self-Determination Theory serves as the theoretical framework, emphasizing autonomy, competence, and relatedness as key motivational factors. The protocol describes including various study types and employing a comprehensive search strategy across multiple databases including PubMed, MEDLINE, and ERIC. The protocol for data extraction will categorize different types of LSAs and identify best practices through thematic analysis.

 Article strengths:

 This submission background information and description of the theoretic framework for which the protocol is based in a strength of the protocol. The description of this scoping review protocol proposed methods are thorough and detailed enough to allow for the review to be reproduced by another researcher. A medical librarian was consulted for the development of the search criteria, further strengthening the methods of the protocol, and the proposed usage of the Joanna Briggs Institute methodology and PRISMA-P are another strength.

 Article weaknesses:

 Overall, this protocol is very strong and so I do not see any significant areas for improvement. While reviewing this submission, I did wonder why the authors chose to narrowly limit the review to medical education (both undergraduate and graduate) rather than expand to include other health professions education. Perhaps the addition of the rationale for this exclusion would strengthen this submission.

 Overall, this submission is a well-written protocol for a scoping review that will yield interesting results and make a significant contribution to the medical education literature regarding LSAs.

Is the study design appropriate for the research question?

Yes

Is the rationale for, and objectives of, the study clearly described?

Yes

Are sufficient details of the methods provided to allow replication by others?

Yes

Are the datasets clearly presented in a useable and accessible format?

Not applicable

Reviewer Expertise:

medical education

I confirm that I have read this submission and believe that I have an appropriate level of expertise to confirm that it is of an acceptable scientific standard.

# Reviewer report: Reviewer response for version 1

**DOI:** 10.5256/f1000research.174161.r354585

**Published:** 2025-01-25

**Authors:** Aubrie Swan Sein

**Affiliations:** 1 Columbia Vagelos College of Physicians and Surgeons, New York, NY, USA

This article outlines a protocol for conducting a scoping review to explore the influence of low-stakes assessments (LSAs) on student learning in medical education, using the Joanna Briggs Institute framework for scoping reviews and the PRISMA-P guidelines. The review will focus on identifying the impact of LSAs on learning outcomes, motivation, engagement, and competency development. It incorporates a comprehensive methodology, including an extensive database search, grey literature exploration, and thematic analysis. The results are anticipated to provide insights into the effectiveness of LSAs and inform best practices for their implementation in medical education.

 Article strengths:

 The authors provide a robust background of LSAs and how they can help foster self-regulated learning. The use of Self-Determination Theory as a conceptual framework makes sense to expand our understanding of how LSAs impact student learning as it relates to the constructs of autonomy, competence and relatedness. The proposed methodology is thorough, including its proposal to incorporate both peer-reviewed and grey literature. Acknowledging the role of grey literature ensures a broader understanding of the topic, capturing less formalized but still valuable research findings. The use of established frameworks like PRISMA-P and the Joanna Briggs Institute methodology adds rigor to the review. The article and proposal is well-organized, with clearly defined objectives, inclusion/exclusion criteria, and a detailed plan for data analysis. The focus on LSAs addresses a significant area in medical education, particularly with the increasing emphasis on formative assessment and competency-based education.

 Article areas for opportunity:

 A few things areas out to me as potential opportunities for improvement. Limiting the review to medical education excludes valuable insights from other health professions education fields (e.g., nursing, pharmacy). Broadening the scope could enhance the generalizability of the findings. A brief justification for this decision to exclude other health professions domains would improve clarity. Also, the inclusion criteria mention grey literature but lack detail on what types will be included (e.g., reports, conference proceedings). Providing examples or a more precise definition would strengthen the protocol. Regarding the MeSH terms, while the authors mention terms like "low-stakes assessment" and "formative assessment," adding related the term "assessment for learning" and maybe “assessment as learning” could increase the breadth of relevant studies.

 Overall, this scoping review protocol has the potential to make a significant contribution to the literature on LSAs in medical education, particularly in guiding their effective implementation to support student learning and engagement.

Is the study design appropriate for the research question?

Yes

Is the rationale for, and objectives of, the study clearly described?

Yes

Are sufficient details of the methods provided to allow replication by others?

Yes

Are the datasets clearly presented in a useable and accessible format?

Yes

Reviewer Expertise:

Assessment for learning, academic counseling

I confirm that I have read this submission and believe that I have an appropriate level of expertise to confirm that it is of an acceptable scientific standard.
